# Unique features in the intracellular transport of typhoid toxin revealed by a genome-wide screen

**DOI:** 10.1371/journal.ppat.1007704

**Published:** 2019-04-05

**Authors:** Shu-Jung Chang, Sheng Chih Jin, Xuyao Jiao, Jorge E. Galán

**Affiliations:** 1 Department of Microbial Pathogenesis, Yale University School of Medicine, New Haven, Connecticut, United States of America; 2 Department of Genetics, Yale University School of Medicine, New Haven, Connecticut, United States of America; Stanford University School of Medicine, UNITED STATES

## Abstract

Typhoid toxin is a virulence factor for *Salmonella* Typhi and Paratyphi, the cause of typhoid fever in humans. This toxin has a unique architecture in that its pentameric B subunit, made of PltB, is linked to two enzymatic A subunits, the ADP ribosyl transferase PltA and the deoxyribonuclease CdtB. Typhoid toxin is uniquely adapted to humans, recognizing surface glycoprotein sialoglycans terminated in acetyl neuraminic acid, which are preferentially expressed by human cells. The transport pathway to its cellular targets followed by typhoid toxin after receptor binding is currently unknown. Through a genome-wide CRISPR/Cas9-mediated screen we have characterized the mechanisms by which typhoid toxin is transported within human cells. We found that typhoid toxin hijacks specific elements of the retrograde transport and endoplasmic reticulum-associated degradation machineries to reach its subcellular destination within target cells. Our study reveals unique and common features in the transport mechanisms of bacterial toxins that could serve as the bases for the development of novel anti-toxin therapeutic strategies.

## Author summary

Typhoid toxin is an important virulence factor for the human pathogen *Salmonella* Typhi, the cause of typhoid fever. This toxin is composed of a pentameric “B” subunit linked to two enzymatic “A” subunits, resulting in an unusual A2B5 configuration. The B subunit targets the toxin’s enzymatic activities by interacting with specific surface receptors. Once internalized, the toxin must be transported to its final subcellular destination by specific transport mechanisms. Here we have used a multidisciplinary approach to define the details of the intracellular transport mechanisms utilized by typhoid toxin. Through a genome-wide screen, we found that typhoid toxin utilizes components of the retrograde transport cellular machinery to arrive to the endoplasmic reticulum, from where it is transported to the cell cytosol by the endoplasmic reticulum-associated degradation pathway. By comparing typhoid toxin’s transport pathway with the transport mechanisms utilized by other toxins we have defined unique a common components that transport these toxins to their cellular destinations. These studies may provide the based for the development of novel anti-toxin therapeutic strategies.

## Introduction

Typhoid toxin is a unique virulence factor for the typhoidal *Salmonella enterica* serovars Typhi and Paratyphi [1–4], the cause of typhoid fever in humans, a systemic disease that remains a major global public health concern [5–9]. When administered to experimental animals, typhoid toxin can reproduce many of the pathognomonic acute symptoms of typhoid fever [1]. The architecture of typhoid toxin is unusual among member of the AB5-toxin family in that it is composed of two enzymatic A subunits, PltA and CdtB, linked to a single pentameric B subunit, PltB [1]. CdtB is a deoxyribonuclease, which causes DNA damage and cell cycle arrest in intoxicated cells, while PltA is an ADP ribosyl transferase with as of yet unidentified targets.

The biology of typhoid toxin is uniquely adapted to the intracellular lifestyle of *Salmonella*. In fact, the toxin is only expressed by intracellularly localized bacteria [2, 4, 10], and after its secretion into the lumen of the *Salmonella*-containing vacuole by a specific protein secretion system [11], it is packaged into vesicle carrier intermediates and exported to the extracellular space [2, 12]. Once exported, typhoid toxin can target a variety of cells by engaging specific cell surface receptors [1]. The autocrine and paracrine pathways are consequently the only mechanism by which the toxin can reach its targets after its interaction with cell surface receptors [2]. Therefore, *S*. Typhi-infected cells potentially lacking receptors for the toxin would not be susceptible to intoxication although they would be competent to harbor bacteria to produce it, a mechanistic feature that may be relevant for the toxin’s proposed role during persistent infection [13].

Consistent with the stringent specificity of *S*. Typhi and *S*. Paratyphi for their human hosts, typhoid toxin has adapted to exert its function preferentially in human cells exhibiting exquisite preference for surface glycoproteins sialoglycans terminated in acetyl neuraminic acid, which are preferentially expressed by human cells [1, 14]. How typhoid toxin reaches its cellular targets after receptor binding is currently unknown. Bacterial toxins utilize a variety of mechanisms to gain access to their cellular targets that most often involve the hijacking of specific cellular machinery for their transport within cells. Here we have used a multidisciplinary approach to define the transport pathway of typhoid toxin within human cells. Through a genome-wide CRISPR/Cas9 screen, we have identified cellular components that are required for typhoid toxin transport within cells. This study provides a detailed view of the transport mechanisms that deliver typhoid toxin from the cell surface to its destination within target cells, and identifies cellular components that are unique to the transport of this toxin as well as components that are also exploited for the transport of other bacterial toxins, thus providing the foundation for the development of novel anti toxin strategies.

## Results

### Typhoid toxin is transported to the endoplasmic reticulum by retrograde trafficking prior to its disassembly and translocation to the cell cytosol

**Fig 1:**
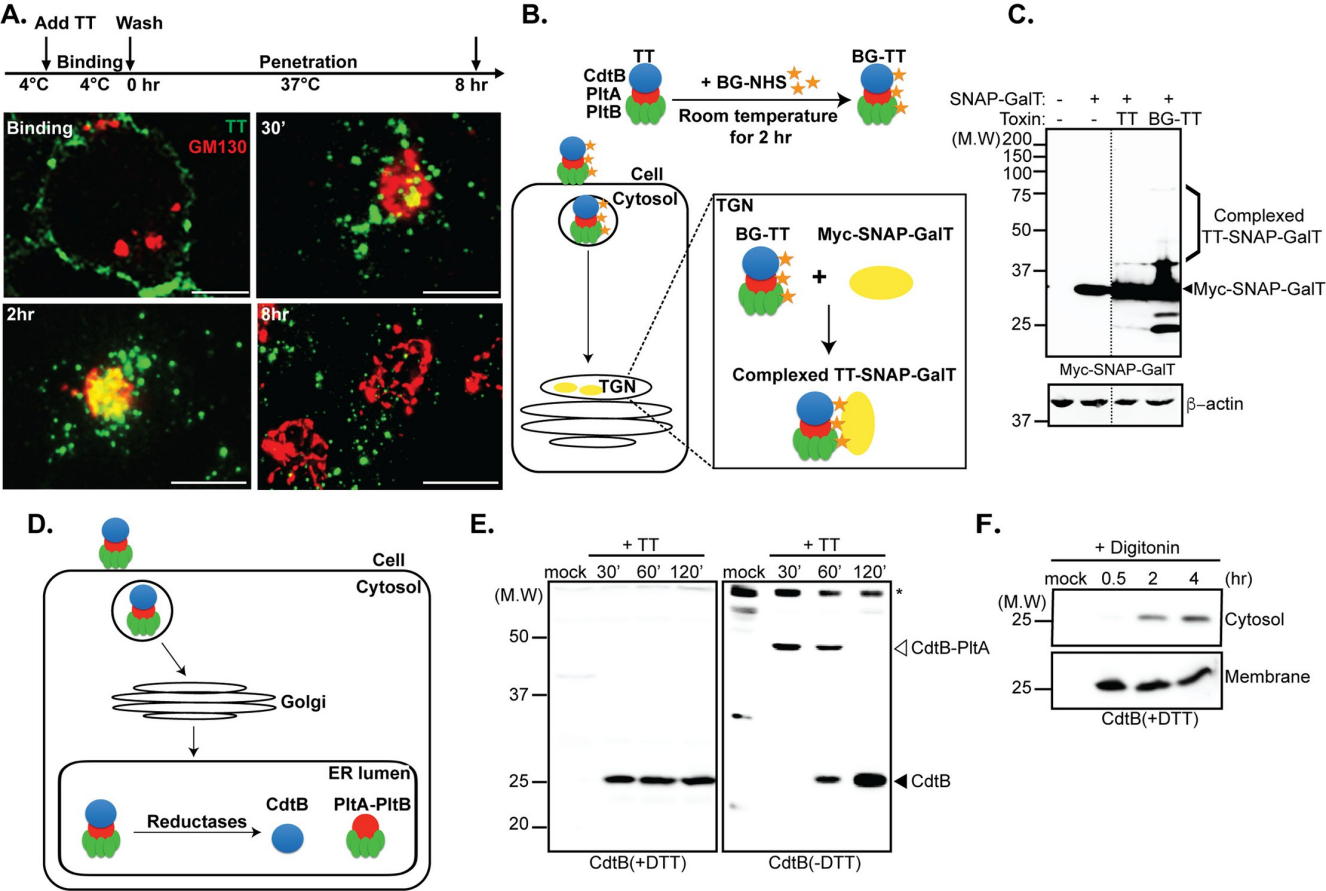
Typhoid toxin traffics to the ER by retrograde transport. (A) Tracking typhoid toxin (TT) transport by immunofluorescence microscopy. HEK293T cells were incubated with Oregon Green-488-labeled typhoid toxin (green) at 4°C for 30 min, washed, and fixed with 4% paraformaldehyde (binding). Alternatively, after 30 min incubation at 4°C, cells were washed, and then switched to 37°C, incubated for 0.5, 2, and 8 hs and fixed as indicated above. Fixed cells were stained with an anti-GM130 antibody (red) and visualized by fluorescence microscopy. Scale bar, 5 μm. (B and C) Typhoid toxin undergoes retrograde transport to the *trans*-Golgi network (TGN). (B) Schematic representation of the assay to detect typhoid toxin transport through the Golgi. (C) HEK293T cells transiently expressing myc epitope tagged SNAP-Galactosyl transferase 1 (Myc-SNAP-GalT) were treated with BG-NHS-labeled (BG-TT) or unlabeled (TT) typhoid toxin for 6 hr at 37°C. BG-labeled toxin molecules that were “captured” by SNAP-GalT formed chimeric protein complexes (indicated as "TT-SNAP-GalT") that were detected by Western blot analysis with an antibody directed to the Myc epitope. Dotted lines indicate places where the experimentally relevant lanes were spliced together (all lanes originate from a single gel). (D and E) Typhoid toxin transport to the endoplasmic reticulum (ER). (D) Schematic representation of the typhoid toxin-disassembly assay in the ER. (E) HEK293T cells were treated with purified typhoid toxin for 30 min at 37°C and lysed at the indicated time points. The mobility of typhoid toxin in SDS-PAGE in the presence or absence of DTT (as indicated) was then analyzed by Western blot with an antibody to CdtB. The positions of CdtB and the CdtB-PltA heteromeric complex are indicated. * denotes the migration of a non-specific cross-reacting protein (F) Typhoid toxin retro-translocation from the ER to the cell cytosol. HEK293T cells were incubated with purified typhoid toxin at 37°C and then harvested at the indicated time points. Cells were selectively permeabilized with digitonin and the presence of typhoid toxin in the cytosolic fraction was detected by Western blot analysis with an antibody to CdtB.

All AB5 toxins whose transport mechanisms have been characterized to date are internalized by receptor-mediated endocytosis and subsequently delivered by retrograde transport first to the Golgi and then to the endoplasmic reticulum, where the holotoxins are disassembled and the enzymatic subunits are translocated to the cytosol [15–19]. To determine whether typhoid toxin follows an analogous uptake pathway, we applied fluorescently-labeled typhoid toxin to cultured cells and examined its fate over time. To synchronize the intoxication process, cultured cells treated with typhoid toxin were incubated at 4°C to allow toxin binding while preventing toxin internalization. After toxin binding cells were switched to 37°C and the fate of the labeled toxin over time was monitored by immunofluorescence microscopy. Consistent with its known interaction with surface glycoproteins [1], typhoid toxin was initially observed bound to the cell surface plasma membrane (Fig 1A). At later (30 min) time points and after the temperature switch, the toxin was observed within fluorescent puncta that most likely represent early endocytic compartments (Fig 1A). Two hours after the temperature shift, the toxin was observed co-localized with a compartment that could be labeled with an antibody to the Golgi marker GM130 (Fig 1A), and later (8 hs after the temperature shift) within puncta spread throughout the cell cytosol.

To verify that typhoid toxin reaches the Golgi, we used a biochemical assay based on the expression of a SNAP-tagged reporter targeted to the Golgi apparatus [20, 21]. The SNAP tag covalently and irreversibly reacts with benzylguanine (BG) therefore BG-labeled proteins can be captured by spatially localized SNAP-tagged reporters where the labeled proteins and the reporter intersect [22] (Fig 1B). We applied BG-labeled typhoid toxin to cells expressing the SNAP-tagged Golgi resident protein GalT and monitored the potential arrival of the toxin to the Golgi over time by a gel mobility assay to detect the typhoid toxin-SNAP-GalT (TT-SNAP-GALT) complex. We found that after treating cells with BG-labeled typhoid toxin we were able to detect the presence of a BG-typhoid toxin-SNAP-tagged complex as demonstrated by the presence of a CdtB band with a shifted mobility (Fig 1C). These results showed that after its internalization, typhoid toxin is delivered to the Golgi, presumably by its retrograde transport from early endocytic compartments.

The PltA and CdtB enzymatic subunits of typhoid toxin are linked to one another by a disulfide bond between two spatially-coordinated cysteine residues [1]. Consequently, disassembly of the holotoxin complex requires the reduction of this disulfide bridge, which can be monitored by western blot analysis. By analogy to other AB5 toxins, reduction of the disulfide bridges should occur upon the toxin’s arrival to the endoplasmic reticulum, most likely mediated by resident disulfide reductases [23]. We therefore reasoned that the reduction of the disulfide bond that tethers CdtB to PltA could serve as a reporter for the arrival of typhoid toxin to the endoplasmic reticulum (Fig 1D). We incubated cultured cells with typhoid toxin at 37°C and the integrity of the disulfide bond that tethers CdtB to PltA over time was monitored in host cell lysates using SDS-PAGE in the presence or absence of a reducing agent, and western blot analysis with an antibody to CdtB (Fig 1E). Early after switching the toxin-treated cells to 37°C (30 min) we detected CdtB with a mobility corresponding to a molecular weight consistent with a CdtB-PltA complex (Fig 1E). In the presence of DTT, the observed mobility of CdtB corresponded to its predicted molecular weight, thus confirming that the slower migrating species observed in the absence of the reducing agent corresponded to the CdtB-PltA complex. Starting at 60 minutes after switching the treated cells to 37°C, the migration of CdtB indicated that the disulfide bond that tethers it to PltA had been reduced upon its arrival to the ER and by the two-hour time point the CdtB-PltA complex was no longer detectable (Fig 1E). We also investigated whether CdtB was translocated to the cell cytosol by applying a differential membrane permeabilization and fractionation protocol to toxin treated cells. We found that starting at 2 hours after treating cells with typhoid toxin, CdtB could be readily detected in the cytosolic fraction of intoxicated cells, an indication of its translocation from the endoplasmic reticulum (Fig 1F).

Taken together, these results indicate that, similar to other AB5 toxins, typhoid toxin is transported to the endoplasmic reticulum through retrograde traffic, where the holotoxin is disassembled prior to the translocation of its enzymatic subunits to the cell cytosol.

### Genome-wide CRISPR/Cas9 screen identifies cellular factors required for toxicity

**Fig 2:**
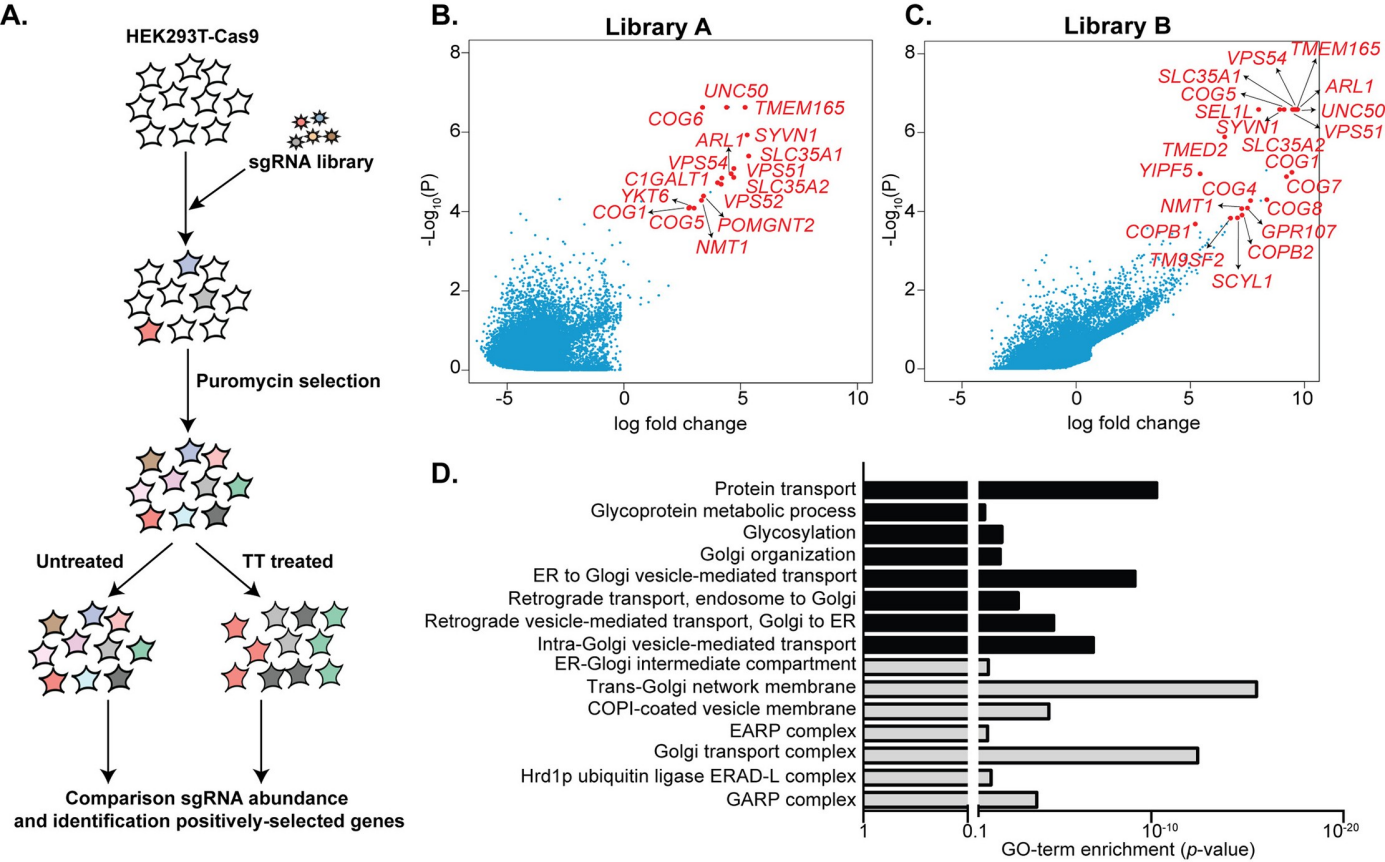
A genome-wide CRISPR/Cas9-mediated gene inactivation screen identifies genes essential for typhoid toxin intoxication. (A) A schematic of the workflow for the screen to identify genes involved in typhoid toxin toxicity. HEK293T cells expressing Cas9 were transduced with a lentiviral library encoding sgRNA targeting human genes as described in Material and Methods. After puromycin selection, cells were split and either mock treated or treated with typhoid toxin. Cells that survived the toxin treatment or that were mock treated were subjected to nucleotide sequence analysis as indicated in Materials and Methods. Sequences were aligned to the reference genome and high-quality reads were analyzed with the MAGeCK algorithm to identify positively-selected genes. (B and C) Scatter plots of effect size (log fold change; x axis) versus *P* value (-log_10_ raw *P*-value; y-axis) for all genes. Three replicates were performed for each sub-library and the MAGeCK algorithm was used to compare treated with untreated cells across replicates for the human GeCKO v.2 sub-library A and B as described in Material and Methods. Inactivation of the genes colored in red conferred resistance to typhoid toxin with a *P*-value cutoff corresponding to 15% FDR. (D) Gene ontology term enrichment analysis of genes whose inactivation conferred toxin resistance. The *P*-values represent the probability of the identified genes to be annotated to a particular GO term relative to all the annotated human genes. GO terms are shown depicting biological processes in black and cellular components in gray.

The results shown above provided a framework for the transport of typhoid toxin after its internalization. However, these studies did not provide insight into the cellular machinery associated with this process. To unravel the mechanisms of toxin transport, we used the CRISPR/Cas9 genome-editing system [24] to conduct a genome-wide screen to identify genes whose disruption conferred resistance to typhoid toxin. It was expected that disruption of toxin transport should protect cells from the toxin’s activity. HEK293T cells constitutively expressing Cas9 were independently transduced with two different sgRNA libraries packaged into lentiviral particles, designed to target each of ~ 20,000 human genes with 3 unique sgRNAs (Fig 2A). Cells transduced with either of the two sgRNA libraries were treated with an amount of typhoid toxin that pilot experiments had determined to result in the death of 80–90% of the treated cells. The premise of the screen was that treatment of the transduced cells with typhoid toxin would enrich for sgRNA-directed mutations that result in cells exhibiting resistance to typhoid toxin due to the inactivation of genes required for intoxication. The pool of cells surviving toxin treatment were collected and compared to parallel untreated samples by deep sequencing of the integrated sgRNAs. To increase the robustness of the screen, all procedures were performed in triplicate and the entire screen was conducted three times. All samples were then compared using the model-based analysis of genome-wide CRISPR/Cas9 knockout (MAGeCK) algorithm [25]. With a false discovery rate (FDR) cutoff of 15%, our screen identified 26 genes whose inactivation led to increased resistance to typhoid toxin treatment, 11 common to both libraries and 5 and 11 unique to libraries A and B, respectively (Fig 2B and 2C and S1 and S2 Tables). Analysis of the identified genes using GO (http://www.geneontology.org) showed a significant enrichment for pathways for protein glycosylation, lipid metabolism, and more prominently, many pathways involved in vesicle transport to the Golgi and the ER (Fig 2D). More specifically, the screen identified genes encoding components of well-characterized multi-protein complexes such as the Golgi-associated retrograde protein (GARP) (*VPS51*, *VPS52*, *VPS53*, *VPS54*) [26] and the conserved oligomeric Golgi (COG) (*COG1*, *COG4*, *COG5*, *COG6*, *COG7*, COG8) complexes [27], as well as components of the ER-associated degradation (ERAD) retro-translocation machinery (*SEL1L* and *SYVN1*) [28, 29]. These pathways presumably captured the different processes involved in TT transport from its uptake to its delivery to the particular subcellular destination where the active subunits exert their function.

### Validation of candidate genes involved in typhoid toxin intoxication

**Fig 3:**
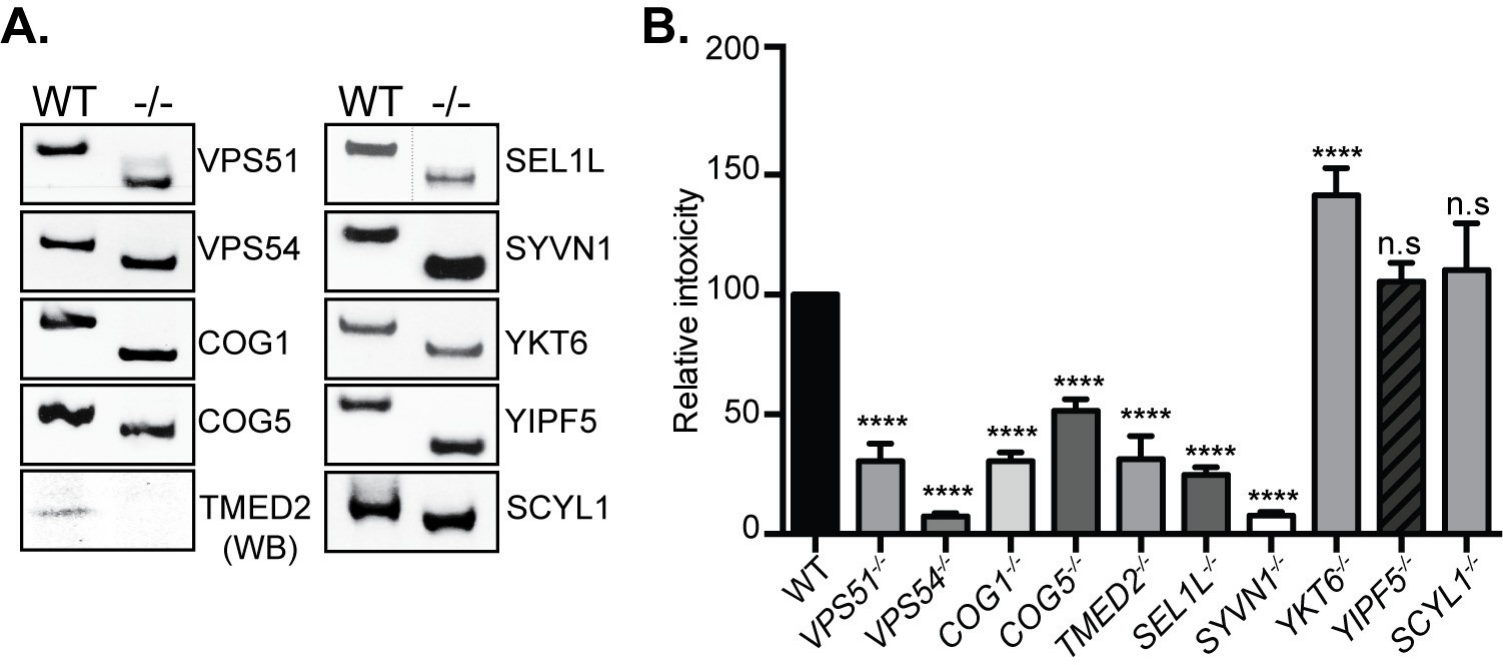
Validation of candidate genes involved in typhoid toxin intoxication. (A) Genotyping of the CRISPR/Cas9-generated knockout cells. Genomic DNA was purified from CRISPR-Cas9 edited HEK293T cell lines and analyzed by PCR with specific primers listed in S3 Table. The TMED2-deficient cell line was examined by Western blot with an anti TEMED2-specific antibody. Dotted lines indicate places where the experimentally relevant lanes were spliced together (all lanes originate from a single gel). (B) Relative toxicity of typhoid toxin in the indicated knockout cell lines after treatment with a serial dilution of purified toxin. The relative toxicity was determined from the percentage of cells at the G_2_M phase fitted by nonlinear regression as indicated in the Materials and Methods. Values, which were normalized relative to wild type (considered 100) are the mean ± SEM of independent determinations. ****p < 0.0001; n. s.: differences not statistically significant; two-tailed Student’s t-test.

To validate the results of the CRISPR/Cas9 screen we generated cell lines individually defective in a subset of the identified genes or pathways (Fig 3A). Specifically, using CRISPR/Cas9 genome editing we generated HEK-293T-cells defective for VPS51, VPS54, COG1, COG5, YKT6, TMED2, YIPF5, SEL1L, SYVN1, YKT6, YIPF5, and SCYL1 and tested the resulting cell lines for their susceptibility to typhoid toxin (Fig 3A and 3B). To assay for typhoid toxin toxicity we examined the ability of the toxin to stimulate G2/M cell cycle arrest in intoxicated cells as a consequence of DNA damage inflicted by its CdtB subunit (Fig 3B). We found that removal of VPS51 and VPS54, two components of the GARP complex [26], confer significant resistance to typhoid toxin. Similarly, removal of the members of the COG complex, COG1 or COG5, which are involved in Golgi trafficking [27], as well SEL1L [28] and SYVN1 [29], which are critical components of the ER-associated degradation (ERAD) pathway, also resulted in significant resistance to intoxication. TMED2, also identified in our screen, is a member of the p24 protein family, which has been implicated in vesicle traffic between the ER and Golgi complex [30, 31]. Consistent with the results of the screen, we found that inactivation of TMED2 conferred significant resistance to typhoid toxin (Fig 3B). In contrast, inactivation of YKT6, YIPF5, or SCYL1, which were also identified in our screen, did not increase resistance to typhoid toxin suggesting that these proteins may not be involved in typhoid toxin transport (Fig 3B). In fact, inactivation of YKT6 appeared to sensitize cells to typhoid toxin. It is unclear why these mutations may not lead to toxin resistance in the context of stable cell lines but we hypothesize that under these conditions, inactivation of these genes may lead to compensatory changes that may facilitate toxin transport through alternative pathways as YKT6, YIPF5, or SCYL1 have been implicated in retrograde transport [32] [33]. Taken together, these findings validate the results of our screen and implicate components of the retrograde and Golgi transport, and ERAD machinery in the transport of typhoid toxin.

### Typhoid toxin transport to the Golgi

**Fig 4:**
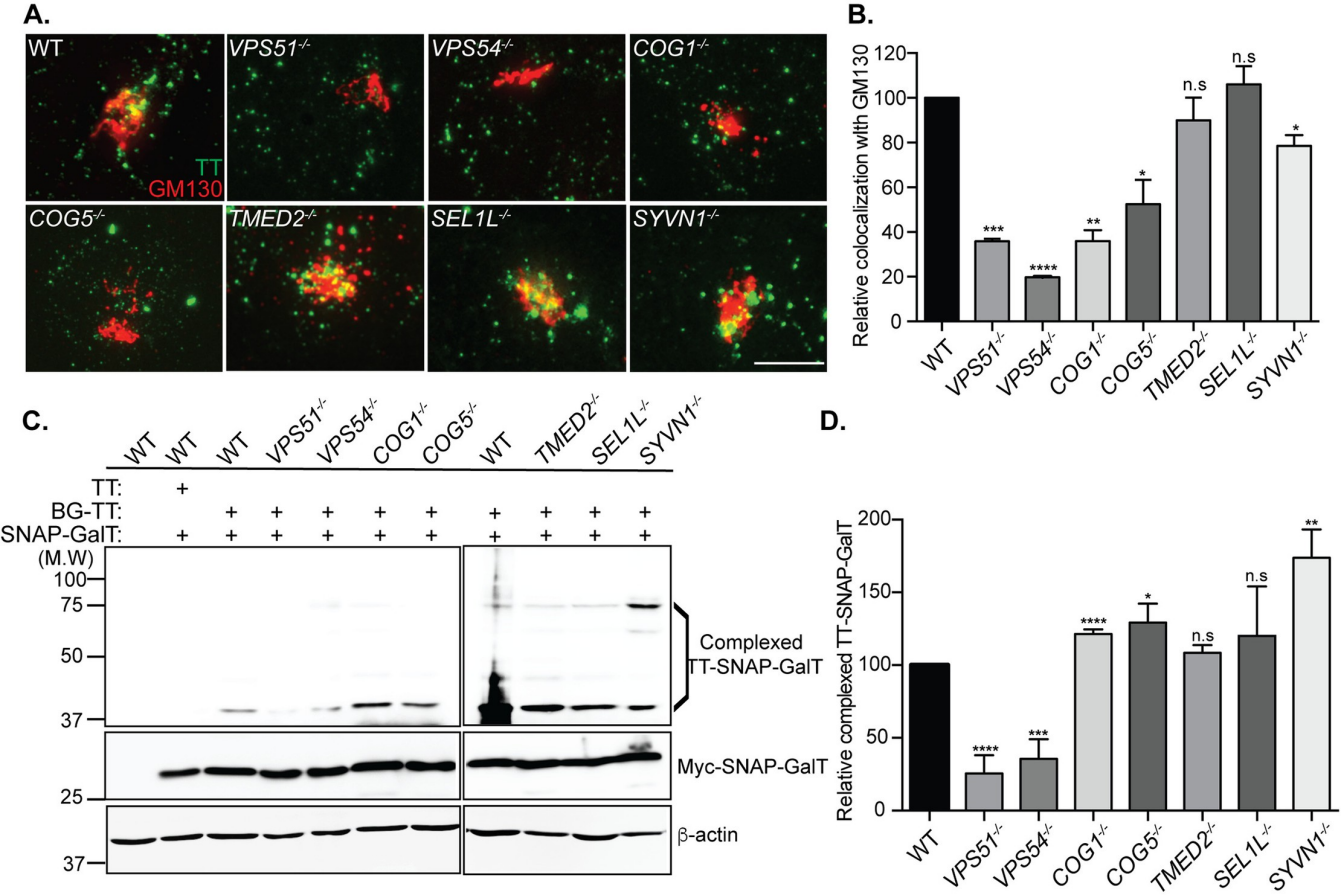
Characterization of typhoid toxin trafficking to the TGN in CRISPR/Cas9-edited cell lines. (A and B) Co-localization of typhoid toxin with the Golgi marker GM130. (A) Wild-type and the indicated knockout cell lines were treated with Oregon-488 labeled typhoid toxin (green) and 2 hours after toxin treatment, cells were stained with an anti-GM130 antibody (red) as described in Material and Methods. Scale bar, 5 μm. (B) The co-localization between typhoid toxin and GM130 was determined as described in Material and Methods. Values represent the relative co-localization (normalized to wild type) and are the mean ± SEM of three independent experiments. ****p < 0.0001, ***p < 0.001, **p < 0.01, and *p < 0.05; two-tailed Student’s t-test. (C and D) Typhoid toxin Golgi localization determined by SNAP-capture. (C) Cells expressing Myc-epitope tagged SNAP-GalT (Myc-SNAP-GalT) were incubated with BG-labeled typhoid toxin for 6 hr and subsequently analyzed by Western blot with an anti-Myc antibody to detect typhoid toxin/SNAP-GalT chimeric protein complexes (TT-SNAP-GalT) and anti β-actin antibody as a loading control. The migration position of the uncomplexed (Myc-SNAP-GalT) and toxin complexed GalT-SNAP (TT-SNAP-GalT) are indicated. (D) Relative amounts of TT-SNAP-GalT quantified from the blots as indicated in Materials and Methods. Values represent the relative intensity of all bands associated with TT-SNAP-GalT (normalized for loading and relative to the values of wild type, which were considered 100) and are the mean ± SEM of 3 independent determinations. ****p < 0.0001; ***p < 0.001; *p < 0.05; n. s.: differences not statistically significant; two-tailed Student’s t-test.

We next sought to investigate the specific contribution of the pathways identified in the screen in the transport of typhoid toxin to the Golgi. We treated cells deficient in specific transport pathways with fluorescently labeled typhoid toxin and examined its co-localization with the Golgi marker GM130. We found that cells deficient in the GARP complex components VPS51 and VPS54 exhibited significantly reduced toxin co-localization with GM130 relative to wild-type cells (Fig 4A and 4B). In contrast cells deficient in TMED2, SEL1L, and SYVN1 exhibited equivalent levels of toxin co-localization with GM130 to those observed in wild type (Fig 4A and 4B). Cells deficient in the COG complex components COG1 and COG5 showed an intermediate phenotype with some reduction in the level of toxin co-localization with GM130 but not as marked as what was observed in cells deficient in GARP complex components (Fig 4A and 4B). We also applied BG-labeled typhoid toxin to cells expressing the SNAP-tagged Golgi resident protein GalT and monitored the formation of a TT-SNAP-GalT complexes. Consistent with the GM130 co-localization studies, typhoid toxin arrival to the Golgi was drastically reduced in cells deficient in the GARP complex components VPS51 and VPS54 (Fig 4C and 4D). In contrast, cells deficient in COG complex components, which showed reduced but still significant level of toxin-GM130 co-localization, showed equivalent levels of TT-SNAP-GalT complex formation than those observed in wild type cells (Fig 4C and 4D). These observations indicate that, consistent with its proposed function [27], the COG complex may be important for mobilizing typhoid toxin through the Golgi but not for its delivery to the Golgi. Consistent with their predicted function [28, 29], cells deficient in TMED2 and the components of the ERAD machinery SEL1L, and SYVN1 showed no defects in typhoid toxin delivery to the Golgi (Fig 4C and 4D). In fact, inactivation of SYVN1 resulted in a slight increase in the levels of TT-SNAP-GalT complex formation (Fig 4C and 4D). It is possible that alteration in the ERAD pathway may result in a slow-down in transit of typhoid toxin through the Golgi, which could lead to more efficient TT-SNAP-GalT complex formation. None of the defective cells showed any reduction in the levels of toxin binding to the cell surface, indicating that disruption of the different components of the transport pathway did not affect the localization of the sialoglycan-containing surface molecules that serve as toxin receptors (S1 Fig). Taken together, these results indicate that the GARP complex is required for the endosome-to-Golgi transport of typhoid toxin while the COG complex most likely contributes to its transport within the Golgi apparatus. These findings are entirely consistent with the involvement of these molecules in vesicle transport.

### TMED2 participates in typhoid toxin transport from the Golgi apparatus to the ER

**Fig 5:**
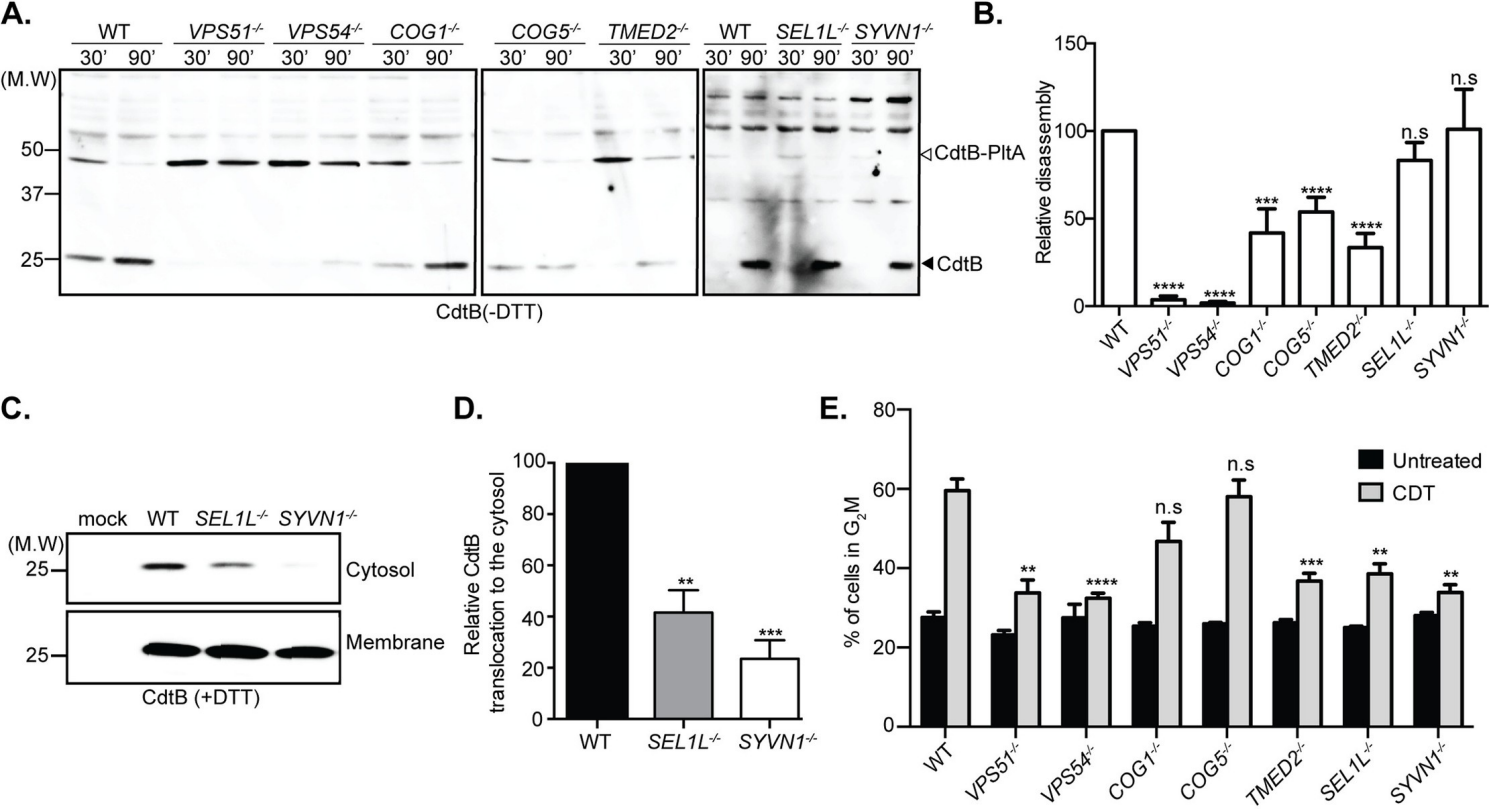
Characterization of typhoid toxin trafficking to the endoplasmic reticulum and cytosol in CRISPR/Cas9-edited cell lines. (A) Wild-type and knockout cells lines were treated with purified typhoid toxin and at the indicated time points, typhoid toxin was recovered from cell lysates by affinity chromatography and analyzed by western blot with an anti toxin antibody as indicated in Materials and Methods. (B) Proportion of typhoid toxin that underwent disassembly as a consequence of its arrival to the ER determined as indicated in Materials and Methods. Values represent the mean ± SEM of 3 independent determinations. ****p < 0.0001, ***p < 0.001; n. s.: differences not statistically significant; two-tailed Student’s t-test. (C) Presence of typhoid toxin in the cytosolic fractions (after retro-translocation) in the indicated knockout cells. Cells were incubated with purified typhoid toxin and fractionated to examine the amount of typhoid toxin in the cytosolic fractions by western blot analysis. (D) Quantification of the relative amount of typhoid toxin in the cytosolic fraction. Values represent the mean ± SEM of three independent experiments. ***p < 0.001, and **p < 0.01; two-tailed Student’s t-test. (E). Toxicity of cytolethal distending toxin in defective cell lines. The parent wild type (WT) and the indicated knockout cell lines were treated with 5 μg of *C*. *jejuni* CDT for 48 hr and subjected to flow cytometric cell cycle analysis. Values are the mean ± SD of five independent experiments. ***p < 0.001, **p < 0.01; n. s.: differences not statistically significant; two-tailed Student’s t-test.

The CdtB and PltA subunits of typhoid toxin are tethered by a disulfide bond that must be reduced prior to their translocation from the ER to the cell cytosol (See Fig 1D and 1E), a process that must be mediated by ER-resident reductases. We therefore tested the disassembly of typhoid toxin in the different defective cell lines as a surrogate assay for its arrival to the ER. We found that consistent with their role in the endosome-to-Golgi transport, cells defective in the GARP complex components Vps51 and Vps54 showed a significant defect in toxin processing as shown by the significant proportion of fully assembled toxin remaining in these cells, an indication of the failure of the toxin to arrive to the ER (Fig 5A and 5B). Like cells defective in the GARP complex, cell lines defective in COG1 and COG5 also showed a defect in toxin transport to the ER (Fig 5A and 5B). As these cells showed reduced but not abolished toxin co-localization with the Golgi marker GM130 (Fig 4A and 4B) and wild type levels of the formation of the TT-SNAP-GALT complex (a reporter for typhoid toxin’s arrival to the Golgi) (Fig 4C and 4D), these results are consistent with the notion that the COG complex is involved in typhoid toxin intra-Golgi transport. Cells defective in the ERAD components SEL1L and SYVN1 did not show defects in typhoid toxin processing indicating that transport to the ER is unaffected in these cell lines (Fig 5A and 5B). However, cells defective in TMED2 showed a marked defect in typhoid toxin disassembly (Fig 5A and 5B). Since these mutant cells showed no measurable defect in the ability of typhoid toxin to arrive to the Golgi (Fig 4A–4D), these results indicate that TMED2 is specifically required for the Golgi-to-ER toxin transport. Although TMED2 has been implicated in vesicle traffic between the ER and Golgi complex [30, 31, 34], it has not been previously reported to be involved in the retrograde transport of AB5 toxins, therefore these findings revealed unique properties in the intracellular transport mechanisms of typhoid toxin.

### Typhoid toxin translocation from the ER to the cytosol requires components of the ERAD pathway

Upon trafficking to the ER, the typhoid toxin A subunits PltA and CdtB dissociate from their B subunit prior to their translocation to the cell cytosol (Fig 1D and 1E). CdtB, which possess a nuclear localization signal, must then be transported from the cytosol to the nucleus where it exerts its function. Our screen identified SEL1L and HRD1 (SYVN1), two components of the endoplasmic-reticulum-associated protein degradation (ERAD) pathway [28, 35], as required for intoxication (Fig 2A–2D). The observation that typhoid-toxin-transport to the ER is unaffected in cell lines deficient in these ERAD components (Fig 5A and 5B) suggested that SEL1L and HRD1 might be involved in the translocation of typhoid toxin components from the ER to the cytosol. To examine this possibility we used a selective permeabilization protocol to probe for the presence of the typhoid toxin subunit CdtB in the cell cytosol. We found that the levels of CdtB in the cytosol of the SEL1L and HRD1 deficient cells was significantly reduced (Fig 5C and 5D), indicating that, similar to other AB5 toxins, typhoid toxin usurps the ERAD pathway for its retrotranslocation to the cell cytosol.

### Typhoid toxin and cytolethal distending toxin transport involves common and distinct cellular components

Cytolethal distending toxin (CDT) is encoded by several pathogenic bacteria including *C*. *jejuni*, some serovars of *Salmonella enterica*, and some pathogenic isolates of *E*. *coli* [36, 37]. It is composed of three subunits, CdtA and CdtC, which serve as its heterodimeric B subunit, and CdtB, which acts as its single A subunit and is a close homolog of typhoid toxin’s CdtB. In fact, *in vitro* experiments have shown that the similarity is such that CdtB from CDT can form a functional complex with PltA and PltB if a Cys residue is added to form the disulfide bond that links it to PltA [38]. In cultured cells, CDT and typhoid toxin shared the ability to stimulate cell cycle arrest due to DNA damage [4, 39]. Like typhoid toxin, CDT is also delivered to cells via retrograde transport mechanisms [40]. However, the specific details of its transport pathway are incompletely characterized. Since typhoid toxin and CDT do not share the same surface receptors, we hypothesized that at least some aspects of their transport mechanism may differ. To identify potentially unique specific aspects in the retrograde transport of these toxins, we examined the susceptibility to CDT of cell lines carrying inactivating mutations in genes involved in typhoid toxin transport. We found that cells deficient in the GARP complex components Vps51 and Vps54, or in TMED2 showed resistance to CDT intoxication, an indication of similarities in the retrograde transport from endosomal compartments as well as from the Golgi to the ER (Fig 5E). Similarly, cells deficient in ERAD components (i. e. SEL1L and HRD1) showed resistance to CDT indicating that, as previously reported [40], CDT utilizes this machinery for its retrograde transport to the cytosol (Fig 5E). In contrast to typhoid toxin, however, cells deficient in the COG complex components COG1 and COG5 were found to be susceptible to CDT intoxication indicating differences in some aspects of the intracellular transport of these toxins (Fig 5E). Taken together, these findings revealed common and unique features in the transport mechanism responsible for the traffic of typhoid toxin and CDT to their cellular destinations.

## Discussion

Typhoid toxin is unique in that, prior to reaching its host cell targets, it must traffic within the cell in opposite directions: 1) after its synthesis within the *Salmonella*-containing vacuole it must be transported to the extracellular space hijacking elements of the cell’s exocytic machinery [2, 41]; and 2) after its transport to the extracellular space, it must enter and traffic within target cells to reach its cellular targets. Here, we have dissected the second of these trafficking events and identified cellular machinery that transports typhoid toxin from the cell surface to its final destination within the intoxicated cells.

To reach their targets within cells, bacterial toxins generally must traverse multiple membrane barriers to gain access to the cell cytosol. These crucial steps involve a variety of strategies, which will ultimately determine the toxin’s transport pathway. While some toxins reach the cell cytosol by directly traversing the cell’s plasma membrane [42, 43], others do so from within various endosomal compartments [19, 44]. Most AB5 toxins gain access to the cell cytosol by hijacking machinery from the ERAD pathway, whose normal function is to remove misfolded proteins from the ER so that they can be transported to the cytosolic proteasome for degradation [19, 45]. Therefore to reach their translocation site, AB5 toxins must be transported from the plasma membrane to the endoplasmic reticulum through a process collectively referred to as retrograde transport. We found that after its receptor-mediated uptake, typhoid toxin follows this overall retrograde transport pathway to the ER. In this compartment, typhoid toxin is disassembled after the reduction of the disulfide bond that tethers its PltA and CdtB enzymatic subunits together, so that they can be individually translocated to the cell cytosol.

**Fig 6:**
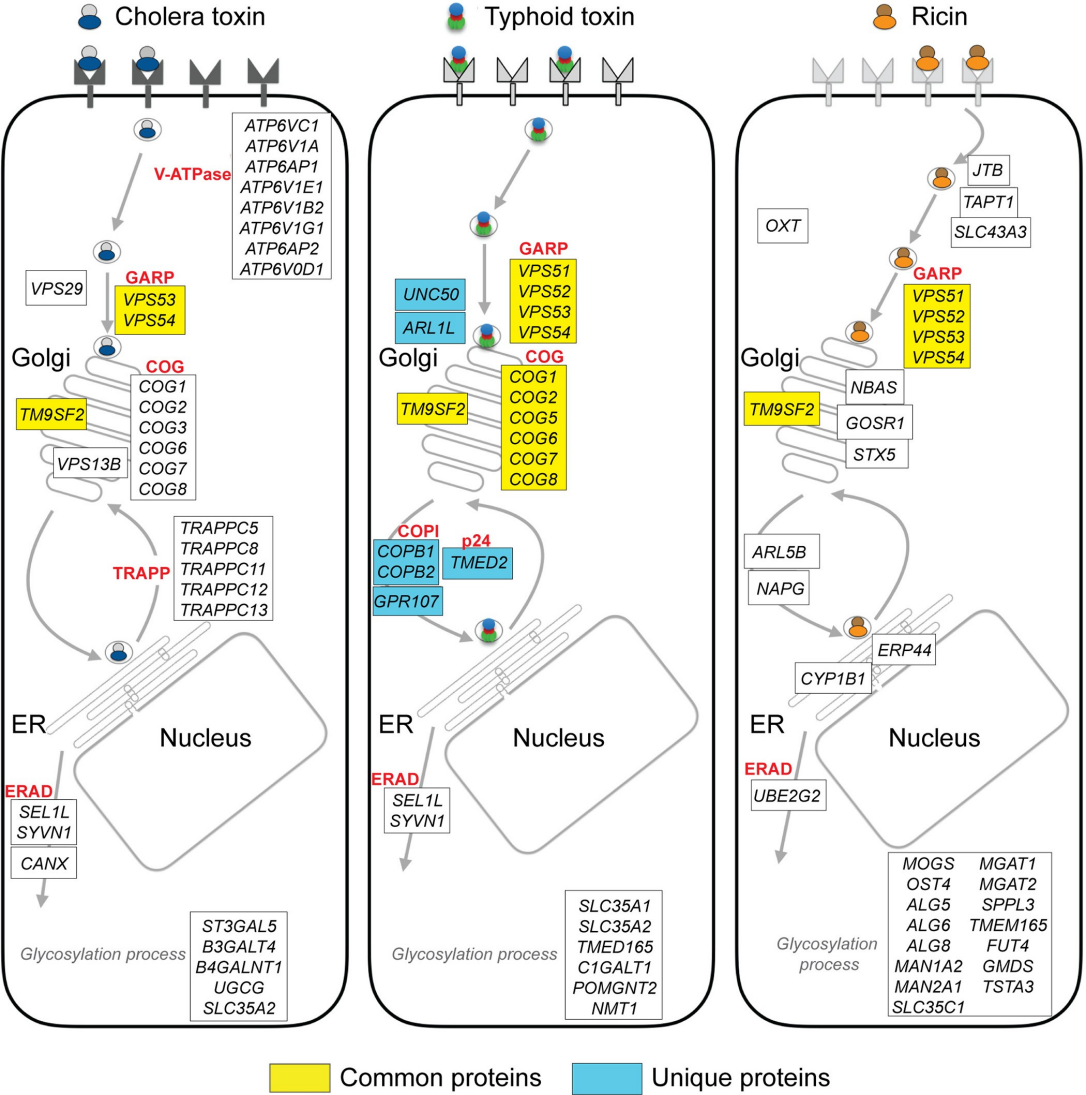
Common and distinct toxin transport pathways revealed by genome wide screens. Boxes indicate known physical complexes. Proteins involved in the transport of all the indicated toxins are depicted in yellow while proteins uniquely involved in typhoid toxin transport are indicated in blue. The trafficking models of cholera toxin and ricin are summarized from Gilbert’s et al. [50] and Tian et al. [51], respectively.

To gain insight into the cellular machinery involved in the transport of typhoid toxin, we carried out a genome-wide CRISPR/Cas9 screen in cultured human cells for genes whose inactivation confer resistance to typhoid toxin. As defects in toxin transport should lead to resistance to intoxication, it was expected that the identity of at least some of these genes should provide a road map for the typhoid toxin transport pathway. This analysis was followed by a more detailed analysis of a selected group of genes, which allowed us to obtain a comprehensive view of typhoid toxin transport (Fig 6).

To gain access to cells, typhoid toxin must first recognize specific acetyl neuraminic acid-terminated sialoglycans on surface glycoproteins or gangliosides [1, 14]. This receptor redundancy most likely results in multiple entry routes, which presumably leads to multiple initial sorting events. This is reflected in our genetic screen in that it did not identify genes that could be assigned to these early events in typhoid toxin transport. For example, although some toxins require clathrin for their internalization [46–48], we found no evidence for clathrin involvement in typhoid toxin uptake, neither in our screen (S1 and S2 Tables) nor by directly targeting clathrin with CRISPR/Cas9-mediated genome editing (S2 Fig). These potentially redundant transport pathways likely converge downstream probably at the level of the Golgi-associated retrograde protein (GARP) complex. Indeed, our screen identified all the components (VPS51-Vps54) of the GARP complex as playing a central role in typhoid toxin transport. The GARP complex is a vesicle-tethering factor that participates in retrograde transport by facilitating the fusion of early and late endosomes-derived vesicle carriers with the Golgi [26, 49]. Therefore this complex is positioned to orchestrate the transport of typhoid toxin to the Golgi regardless of its internalization route. The GARP complex has been shown to play a central role in the transport of other AB5 toxins, therefore emerging as a major hub in the retrograde transport of bacterial toxins [50, 51]. Consistent with the requirement of the GARP complex for typhoid toxin transport, our screen also identified Arl1, a GTPase that is thought to play a regulatory role for GARP complex function [52] (Fig 2 and S1 and S2 Tables). It is likely that the GARP complex works in conjunction with additional proteins to facilitate the transport of typhoid toxin from early and late endosome to the Golgi. Candidate proteins identified in our screen (Fig 2 and S1 and S2 Tables) that may work in concert with GARP include UNC50, which has been implicated in a similar function for Shiga toxin [53], and COPB1 and COPB2, which are components of the COP1 coat involved in vesicle transport [54], further supporting the involvement of this traffic machinery in typhoid toxin retrograde transport.

Our screen identified several genes encoding proteins or protein complexes involved in Golgi transport, notably all but one of the 8 components of the conserved oligomeric Golgi (COG) complex (COG1, COG2, COG4, COG5, COG6, COG7 and COG8) [27, 55] (Fig 2 and S1 and S2 Tables). The COG complex functions as a vesicular tether during retrograde intra-Golgi trafficking. Consistent with this function, typhoid toxin was unable to reach the ER in cultured cell lines engineered to be deficient in specific COG complex components (Fig 5A and 5B). However, typhoid toxin was able to reach the TGN in these cells as demonstrated by its ability to interact with the SNAP-tagged Golgi resident protein GalT (Fig 4C and 4D), indicating that, as predicted by its function, the COG complex may coordinate typhoid toxin transport through the Golgi. The role of other Golgi resident proteins that our screen determined to be necessary for efficient intoxication, such as solute carrier family protein 35A1 and 35A2 (SLC35A1 and SLC35A2) [56], transmembrane protein 65 (TMEM165) [57], Core 1 UDP-Galactose:N-Acetylgalactosamine-Alpha-R Beta 1,3-Galactosyltransferase 1 (C1GALT1) [58], and O-Linked Mannose N-Acetylglucosaminyltransferase 2 (POMGNT2) [59] (Fig 2 and S1 and S2 Tables) is less clear as these proteins are involved in glycosylation reactions and therefore may indirectly alter typhoid toxin transport. The same may apply to N-myristoyl transferase-1 (NMT1), which has been shown to alter Golgi transport by affecting the Golgi membrane/cytosol partitioning of ADP-ribosylation factor (Arf) proteins [60].

We found that the resident Golgi protein TMED2 plays an essential role in typhoid toxin’s transport from the Golgi to the ER (Fig 5A and 5B). This result is noteworthy, as TMED2 has not been previously implicated in toxin transport. These findings are also consistent with the proposed role of this Golgi-resident p24 protein family member in the transport between the *cis*-Golgi network and the ER [30, 31, 34]. TMED2 possess a large luminal N-terminus and a short cytoplasmic C-terminal tail at the cytosol. Via its cytoplasmic tail, TMED2 interacts with ADP-ribosylation factor 1 (ARF1), COPI, and COPII subunits, which suggest that TMED2 can act as cargo receptor and coat protein in vesicle transport. Consistent with this notion, our screen identified the COP components COPB1 and COPB2 as required for typhoid toxin intoxication.

Our results clearly implicate the endoplasmic reticulum associated degradation (ERAD) pathway in the translocation of typhoid toxin from the ER to the cell cytosol (Fig 5C and 5D). The ERAD is involved in the transport of misfolded proteins from the ER to the cytosol for their subsequent delivery to and degradation by the proteasome [23, 61]. There are different mechanisms by which proteins are translocated via this pathway, which are largely dependent on whether the misfolded proteins are located in the lumen of the ER, within the ER membrane, or on the cytosolic side of the ER membrane. Our screen identified the E3 ubiquitin ligase HRD1 (SYVN1), which forms a channel through which misfolded proteins and presumably unfolded toxins pass through the ER membrane [62] (Fig 5C and 5D). Other postulated components of the translocation machinery include HRD3, USA1, DER1, and YOS9, none of which were identified in our screen. However, our screen identified suppressor/enhancer of Lin-12-like (Sel1L) as essential for typhoid toxin translocation from the ER (Fig 5C and 5D). Sel1L has been shown to be an integral component of the HRD1 complex, playing an essential role in the transport of a subset of ERAD substrates including cholera toxin [50, 63].

Overall, our screen identified several components that previous studies have implicated in the retrograde transport of other bacterial toxins, including the GARP, COG, and various components of the core ERAD pathway (Fig 6). Therefore these core toxin transport components can be viewed as hubs that are central for toxin transport and thus can potentially serve as targets for the development of novel broadly-acting antitoxin strategies. However, our screen also identified proteins that, although implicated in various transport functions, including the transport of other bacterial toxins, their mechanism of action is less well understood. These include Golgi-localized G protein couple receptor 107 (GPR107) and Transmembrane 9 Superfamily Member 2 (TM9SF2) (Fig 2 and S1 and S2 Tables), which have been implicated in the transport *Pseudomonas aeruginosa* exotoxin A, CDT, and Shiga toxins [40, 51, 64, 65]. The study of toxin transport mechanisms could thus provide major insight into the activity of these proteins, which have also been implicated in human pathologies including periodontal disease, viral infections, and cancer [66–70].

Our study revealed common features between the toxin transport mechanism of typhoid toxin and *C*. *jejuni* CDT (Fig 5E). This observation is relevant since both toxins share the active subunit in whose activity this genetic screen was based. However, despite the existence of common core factors involved in toxin transport, it is clear that there are unique features in the transport mechanisms of specific bacterial toxins. For example, our study revealed that while the COG complex components COG1 and COG5 are required for typhoid toxin intoxication, these proteins were dispensable for CDT intoxication. Furthermore, comparison of our results with those of other genome wide screens conducted with other toxins demonstrate that, despite sharing some common core components of the cellular transport machinery, each toxin does exhibit unique aspects in their transport pathways (Fig 6) [50, 51, 65], which may revealed variations in vesicle transport mechanisms that have not yet been captured by more directed cell biological studies. This in turn illustrates the value of the study of bacterial toxins as tools to gain insight into basic cellular functions.

In summary, our study has provided a road map for the transport pathway of typhoid toxin in intoxicated cells. These findings can provide a framework for the development of novel therapeutic strategies to combat typhoid fever and other infectious diseases.

## Materials and methods

### Plasmids, antibodies and reagents

All plasmids used in this study are listed in S4 Table and were constructed using the Gibson strategy [71] and were verified by nucleotide sequencing. Antibodies to Myc (Cell Signaling Technology, Cat. #2276), TMED2 (Santa Cruz Biotechnology, Cat.# sc376458), and GM130 (BD Bioscience, Cat. # 610822) were purchased from the indicated commercial sources. Antibodies to purified recombinant typhoid toxoid were generated by Pocono Rabbit Farm & Laboratory. BG-GLA-NHS (Cat. #S9151) was purchased from New England Biolabs and the Human GeCKOv2 libraries (Cat. #1000000049) and the plasmid psPAX2 (Cat. #12260) were purchased from Addgene.

### Mammalian cell culture and generation of the Cas9 stable cell line

All cell lines were grown in Dulbecco’s modified Eagle medium (DMEM, Gibco) supplemented with 10% fetal bovine serum (FBS) at 37°C with 5% CO2 in a humidified incubator, and were routinely checked for mycoplasma with a Mycoplasma Detection Kit (SouthernBiotech, Cat# 13100–01). To generate cells stably expressing wild-type Cas9 endonuclease (HEK293T-Cas9), HEK293T cells (ATCC) were transduced with lentiviral particles produced from lentiCas9-Blast (Addgene, #52962) and selected for blasticidin resistance.

### LentiCRISPR virus production and CRISPR/Cas9 library transduction

The preparation of lentiCRISPR library A and B was carried out as described previously [24]. Briefly, HEK293T cells were seeded on thirty-five 100 × 20 mm tissue culture dishes and grown to 30% confluence. Each plate was then transfected with 7 μg of the human CRISPR Knockout Pooled Library DNA (GeCKO v.2 library), and 3.5 and 5 μg of pVSVg and psPAX2 plasmid DNA, respectively, using Lipofectamine 2000 with PLUS^TM^ reagent (Life Technologies). After 5hr, the media was changed to DMEM supplemented with 10% FBS and 1% BSA (Sigma). The culture media was pooled, centrifuged at 3,000 rpm for 10 min at 4°C to pellet cell debris, and supernatants were filtered through 0.45 μm low-protein-binding membranes. To concentrate the pooled library, viral particles were centrifuged at 24,000 rpm for 2 hr at 4°C and pellets resuspended in DMEM with 1% BSA for further use in cell transduction. The viral libraries were titered as follows. HEK293T-Cas9 cells were seeded in 6-well plates and transduced with varying amounts of the viral preparations in the presence of polybrene (8 μg/ml). The 6-well plates were centrifuged at 2,000 rpm for 2 hr at 37°C, and the infection media replaced with fresh media. After 24 hr, cells were detached using trypsin and split into duplicate wells with or without puromycin (0.5 μg/ml). After 1–2 days, cells were counted to calculate the percentage of transduction. Large-scale transduction of 4 × 10^7^ cells was carried out in the same manner and incubated with media containing puromycin for 7 days.

### Typhoid toxin resistance screen

HEK293T-Cas9 transduced with the lentivirus GeCKOv2 libraries targeting human genes were grown on 6-well plates and subjected to puromycin-resistance selection for 7 days as indicated above. Transduced cells (3 × 10^7^) were then treated with media alone (control group) or 40 ρM of typhoid toxin (typhoid toxin treated group) at 37°C for 60 min and changed to normal culture media. The control group was harvested 2 to 3 days post treatment when cells reached 90% confluence. The surviving cells from the typhoid toxin treated group were harvested 15 days post treatment, re-seeded onto 15 cm dishes and then harvested when they reached 90% confluence. Genomic DNAs from toxin or mock treated cells were purified with Blood & Cell Culture Midi kit (Qiagen). A two-step PCR amplification protocol with Illumina sequencing adapters and sample barcodes was applied as described previously using primers listed in S3 Table. Briefly, DNA fragments containing lentiCRISPR sgRNA sequences were first amplified using primers CRISPR-F1 and R1 (see S3 Table). A second PCR was conducted to attach Illumina adaptors and barcode samples using CRISPR-F2 and a R2 primer contanining a unique barcode. PCR products were separated on 2% agarose gels and extracted with the QIAquick Gel Extraction kit (Qiagen). Samples were sequenced on a HiSeq 2500 (Illumina) at the Yale Center for Genomic Analysis suing CRISPR sequencing primers (see S3 Table). To identify sgRNA sequences, the sequence reads were trimmed for quality and length using the Cutadapt program (http://journal.embnet.org/index.php/embnetjournal/article/view/200). Bowtie v1.1.2 (http://bowtie-bio.sourceforge.net/index.shtml) was then used to align the sequence reads back to a reference file of all sgRNA sequences in Library A or B (provided by Addgene). The MAGeCK algorithm was used to identify positively selected genes in each library separately [25]. A total of 6 independent screens were conducted, 3 for each of the lentiCRISPR libraries.

### Toxin expression and purification

Purification of typhoid toxin and cytolethal distending toxin (CDT) was conducted as described previously [1, 72]. Briefly, the genes encoding typhoid toxin in *Salmonella* Typhi (*pltA/pltB*/6xHis-*cdtB*) or CDT in *Campylobacter jejuni* (*cdtA/cdtC*/6xHis-*cdtB*) were cloned into the pET28a (Novagen) expression vector. *Escherichia coli* strains carrying the different plasmids were grown at 37°C in LB media to an OD_600_ of ~0.6, toxin expression was induced by the addition of 0.5 mM IPTG, and cultures were further incubated at 25°C overnight. Bacterial cell pellets were resuspended in a buffer containing 15 mM Tris-HCl (pH 8.0), 150 mM NaCl, 0.1 mg/ml DNase, 0.1 mg/ml lysozyme, and 0.1% PMSF and lysed by passage through a cell disruptor (Constant Systems Ltd.). Toxins were then purified from bacterial cell lysates through affinity chromatography on a Nickel-resin (Qiagen), ion exchange, and gel filtration (Superdex 200) chromatography as previously described [1, 72]. Purified toxins were examined for purity on SDS-PAGE gels stained with coomassie blue.

### CRISPR/Cas9 gene inactivation in cultured human cells

CRISPR/Cas9-edited cell lines were generated as previously described [12]. Briefly, HEK293T cells were transfected with plasmids encoding the different sgRNA, Cas9 and puromycin resistance genes using Lipofectamine 2000. Transfected cells were then treated with puromycin for selection and isolated clones were further screened by PCR genotyping using the primers listed in S3 Table. At least two independently isolated clones per cell line were characterized for the relevant phenotypes. In all cases, the different cell lines exhibited equivalent phenotypes.

### Cell cycle analysis as a measure of toxicity

Cell-cycle arrest after typhoid toxin intoxication was examined by flow cytometry as previously described [2]. Briefly, cells were collected and fixed overnight with 70% ethanol in DPBS at -20°C. Fixed cells were washed with DPBS and resuspended in 0.5 ml of DPBS containing 50 μg/ml propidium iodide, 0.1 mg/ml RNase A and 0.5% Triton X-100 and incubated for 30 min at 37°C. Cells were then washed with DPBS, filtered, and analyzed by flow cytometry on a BD Accuri C6 flow cytometer. The DNA content of cells was determined using FlowJo (https://www.flowjo.com/). Relative toxicity was determined by measuring the concentration of typhoid toxin resulting in 50% of the treated cells (wild type and CRISPR/Cas9 edited cell lines) in G2/M. Briefly, the different cells were treated with a serial dilution of a typhoid toxin preparation, and the percentage of cells in G2/M was determined by flow cytometry as described above. Values were fitted to an orthogonal polynomial regression of degree 2 to estimate the relationship between toxin concentration and % of cells in G2/M using the R software version 3.4.4 (https://www.r-project.org).

### Typhoid toxin labeling

Purified typhoid toxin was fluorescently labeled with Oregon Green (OG)-488 dye (Invitrogen) according to the vendor’s recommendations. Briefly, purified toxin preparations (1 mg/ml) were incubated with the OG-488 dye in 100 mM bicarbonate buffer for 1 h at room temperature and applied to a size-exclusion chromatography column to separate the toxin from the free dye.

### Typhoid toxin binding and internalization assays

Typhoid toxin binding was assayed by flow cytometry as previously described [1]. Briefly, wild type and CRISPR/Cas9-edited HEK293T cells were seeded in 24-well plates for 24 hr and incubated with 0.2 μg of OG-488-labeled typhoid toxin for 60 min at 4°C. Cells were then fixed with 1% of paraformaldehyde, subjected to flow cytometric analyses on a BD Accuri C6 flow cytometer, and the resulting data were analyzed with FlowJo. To evaluate toxin binding and internalization by immunofluorescence microscopy, wild type and CRISPR/Cas9-edited HEK293T cells were treated with fluorescently-labeled toxin for 30 min at 4°C, washed with PBS twice, and then switched to 37°C for 0.5, 2, and 8 hr. Cells were then fixed with 4% paraformaldehyde, and stained with an antibody directed to the *cis*-Golgi marker GM130 (BD Bioscience) overnight at 4°C, and an Alexa 594-conjugated anti-mouse antibody (Invitrogen) for 1 hr at room temperature. Cells were then observed under Nikon TE2000 fluorescence or a Leica TCS SP6 Confocal microscopes.

### Quantification of typhoid toxin co-localization with a Golgi marker

The co-localization of typhoid toxin with the *cis*-Golgi marker GM130 was quantified by fluorescent microscopy using the Coloc 2 plugin of the open source software ImageJ https://imagej.nih.gov/ij/

### BG labeling of typhoid toxin

Purified typhoid toxin (50 μg) was incubated for 2 hr at room temperature with BG-NHS (New England BioLabs, Cat #S9151S) (20 mM stock solution in DMSO) at a molar ratio of 1:3. The unreacted esters were quenched with 50 mM Tris (pH 8) and excess BG-NHS was removed with an Amicon ultra spin column.

### SNAP-binding assay

Wild type and the different CRISPR/Cas9-edited HEK293T cells (6 X 10^5^ /ml) were seeded on 6-well plates and transfected with a plasmid encoding myc-epitope tagged GalT-SNAP using Lipofectamine 2000. Next day, cells were treated with either 0.5 μg of BG-labeled or unlabeled typhoid toxin, harvested 6 hr after treatment, and cell lysates were analyzed by Western blot with an anti-Myc antibody. The amount of typhoid toxin-SNAP-GalT complex in wild type and the different CRISPR/Cas9 edited cell lines was determined by measuring the densities of all bands associated with this complex (as shown by the shift their molecular weight) using the Image Studio Lite software (Li-COR Biosciences) normalized for loading, relative to wild type, which was given a value of 100.

### Typhoid toxin disassembly assay

Wild type and CRISPR/Cas9-edited HEK293T cells (1 X 10^7^) were seeded on 10 cm dishes and subsequently treated with 100 ng of purified His-tagged typhoid toxin at 37°C for 30 minutes. Cells were washed in DPBS to remove unbound typhoid toxin, incubated in media containing 10% FBS for indicated times, lysed in lysis buffer [(150 mM NaCl, 50 mM Tris-HCl (pH 7.4), 0.5% Triton-100, 1X protease inhibitor cocktail (Roche)] for 30 min at 37°C, and centrifuged at 14, 000 rpm for 15 min. Typhoid toxin from the soluble fractions was recovered by affinity chromatography through a nickel resin (Qiagen) after overnight incubation at 4°C and subsequent elution in 30 μl of an elution buffer containing 200 mM imidazole and 0.15 M Tris-HCl (pH 6.8) for 20 min at room temperature. The protein eluates were analyzed by western blot with a rabbit anti-typhoid toxin antibody and a secondary HRP-conjugated goat anti-rabbit antibody in the presence or absence of DTT. Blots were quantified with Image Studio Lite (Li-COR Biosciences) and the proportion of assembled and dissembled toxin was determined by the ratio of the intensities of the bands corresponding to the CdtB-PltA complex and the CdtB monomer. Relative disassembly was determined by comparing the values to those of wild type, which was considered 100.

### Typhoid toxin retro-translocation assay

Wild type and CRISPR/Cas9-edited HEK293T cells (1 X 10^7^) were seeded on 10 cm dishes and treated with 1 μg of purified His-tagged typhoid toxin for 30 min at 37°C. Lysates were resuspended in 500 μl of HCN buffer containing 50 mM HEPES (pH 7.5), 150 mM NaCl, 2 mM CaCl_2_, 0.04% Digitonin, and 1X of a protease inhibitor cocktail (Roche) for 10 min at 4°C. Cytosolic (soluble) and membrane (pellet) fractions were separated by centrifugation at 14, 000 rpm for 10 min. Pellets were resuspended in 2 x Laemmli buffer and soluble fractions were subjected to nickel affinity chromatography to recover typhoid toxin as described above. The relative amounts of typhoid toxin in the different samples was then assayed by Western blot with an anti toxin antibody using the Image Studio Lite (Li-COR Biosciences) software as described above.

### Quantification and statistical analysis

The *p* values were calculated using a two-tailed, unpaired Student’s *t* test for two group comparisons in GraphPad Prism (GraphPad software). *P* values <0.05 were considered significant. Details of the statistical tests used to evaluate the significance of all observations (including the statistical test, precision and dispersion metrics, the n values used as well as how significance is defined), is provided in the corresponding figure legends. The methods of statistical analysis are also described for individual experimental approaches in the Methods section above.

### Data and software availability

The following software was used in this study: Graphpad Prism (plotting data), Micro-Manager, Slidebook 6, and Leica Application Suite Advanced Fluorescence (image acquisition), Adobe Illustrator & Adobe Photoshop (image preparation), FlowJo (analysis of flow cytometry data), R project (scatter plots of CRISPR screen results), Bowtie v1.1.2 (alignment of the sequence reads), and Image Studio Lite (Li-COR Biosciences) (quantification of the band intensity of western blot).

## Supporting information

S1 Fig Typhoid toxin binding to CRISPR/Cas9 edited cell lines. (A) Different defective and their parent HEK293T cell lines were treated with fluorescently labeled typhoid toxin for 30 minutes at 4°C, washed, and subsequently analyzed by flow cytometry as indicated in the Material and Methods. Values represent percentage of typhoid toxin (TT) binding standardized relative to TT binding to wild type (WT) cells, which was considered to be 100% and are mean ± SD from three independent experiments. Two-tailed Student’s t-tests were performed to determine the statistical significance between wild-type and each deficient cell line. *p < 0.05, **p < 0.01. (B) Visualization of typhoid toxin on HEK 293T and defective cell lines. WT and knockout derivatives were treated with fluorescently labeled typhoid toxin (green) for 30 minutes at 4°C. The cells were then fixed and immunostained with an antibody against the GM130 (red) visualized by Leica SP6 confocal. Scale bar, 5 μm.
(DOCX)

S2 Fig Typhoid toxin toxicity in a clathrin heavy chain (CLTC)-deficient cell line. Wild-type (WT) and CLTC knockout cells were mock treated or treated with serial dilutions of typhoid toxin for 48 hours and subjected to flow cytometric cell cycle analysis. Data are the mean ± SD of three independent experiments. The CLTC-deficient cell line was examined by western blot with a specific antibody. Inset shows the Western blot analysis of the wild type and CLTC-deficient (KO) cell lines for the presence of CltC.
(DOCX)

S1 Table Statistical analysis of CRISPR/Cas9 screen. (XLS)

S2 Table Deep sequencing data of the human GeCKOv2 library. (XLS)

S3 Table The list of primers used in this study. (PDF)

S4 Table Plasmids used in this study. (PDF)
