# Supplementary material for: Unique features in the intracellular transport of typhoid toxin revealed by a genome-wide screen
Source: PLoS Pathog. 2019 Apr 5;15(4):e1007704. doi: 10.1371/journal.ppat.1007704 (PMC6469816; doi:10.1371/journal.ppat.1007704)
Supplement: S1 Fig — (A) Different defective and their parent HEK293T cell lines were treated with fluorescently labeled typhoid toxin for 30 minutes at 4°C, washed, and subsequently analyzed by flow cytometry as indicated in the Material and Methods. Values represent percentage of typhoid toxin (TT) binding standardized relative to TT binding to wild type (WT) cells, which was considered to be 100% and are mean ± SD from three independent experiments. Two-tailed Student’s t-tests were performed to determine the statistical significance between wild-type and each deficient cell line. *p < 0.05, **p < 0.01. (B) Visualization of typhoid toxin on HEK 293T and defective cell lines. WT and knockout derivatives were treated with fluorescently labeled typhoid toxin (green) for 30 minutes at 4°C. The cells were then fixed and immunostained with an antibody against the GM130 (red) visualized by Leica SP6 confocal. Scale bar, 5 μm. (DOCX) [file ppat.1007704.s001.docx]

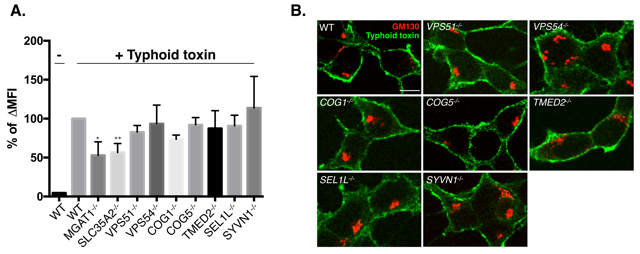


**Supplementary Figure S1.** Typhoid toxin binding to CRISPR/Cas9 edited cell lines. **(A)** Different defective and their parent HEK293T cell lines were treated with fluorescently labeled typhoid toxin for 30 minutes at 4°C and subsequently analyzed by flow cytometry as indicated in the Material and Methods. Values represent percentage of typhoid toxin (TT) binding standardized relative to TT binding to wild type (WT) cells, which was considered to be 100% and are mean ± SD from three independent experiments. Two-tailed Student’s t-tests were performed to determine the statistical significance between wild-type and each deficient cell line. *p < 0.05, **p < 0.01. **(B)** Visualization of typhoid toxin on HEK 293T and defective cell lines. WT and knockout derivatives were treated with fluorescently labeled typhoid toxin (green) for 30 minutes at 4°C. The cells were then fixed and immunostained with an antibody against the GM130 (red) visualized in a Leica SP6 confocal microscope. Scale bar, 5 µm.
