# Supplementary material for: Unique features in the intracellular transport of typhoid toxin revealed by a genome-wide screen
Source: PLoS Pathog. 2019 Apr 5;15(4):e1007704. doi: 10.1371/journal.ppat.1007704 (PMC6469816; doi:10.1371/journal.ppat.1007704)
Supplement: S2 Fig — Wild-type (WT) and CLTC knockout cells were mock treated or treated with serial dilutions of typhoid toxin for 48 hours and subjected to flow cytometric cell cycle analysis. Data are the mean ± SD of three independent experiments. The CLTC-deficient cell line was examined by western blot with a specific antibody. Inset shows the Western blot analysis of the wild type and CLTC-deficient (KO) cell lines for the presence of CltC. (DOCX) [file ppat.1007704.s002.docx]

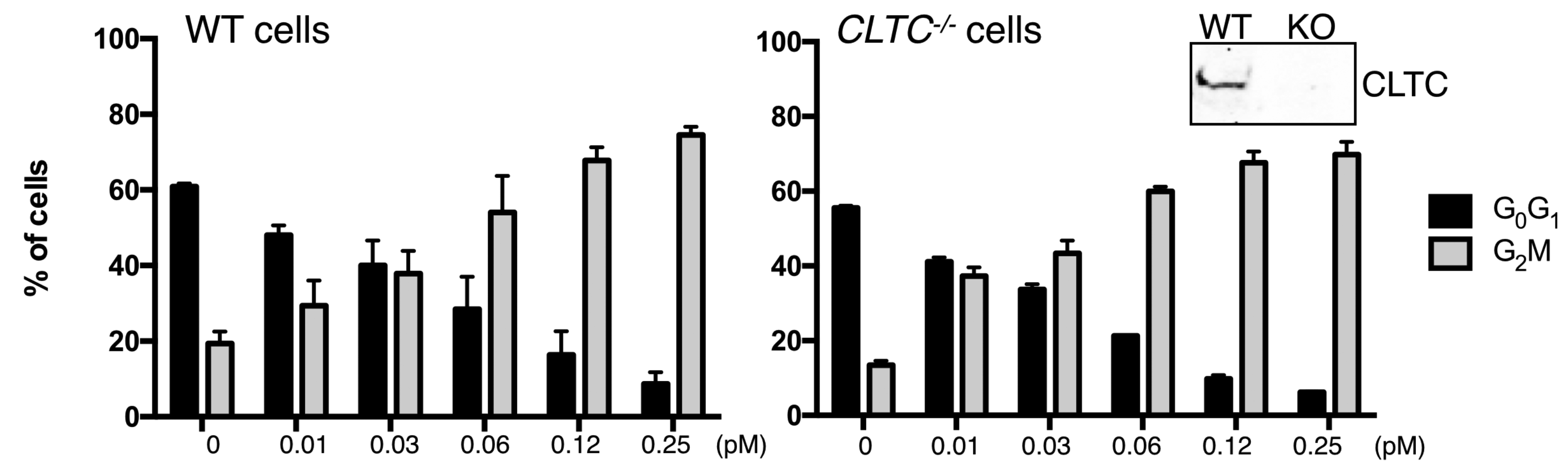


**Supplementary Figure S2. Typhoid toxin toxicity in a clathrin heavy chain (CLTC)-deficient cell line.** Wild-type (WT) and CLTC knockout cells were mock treated or treated with serial dilutions of typhoid toxin for 48 hours and subjected to flow cytometric cell cycle analysis. Data are the mean ± SD of three independent experiments. The CLTC-deficient cell line was examined by western blot with a specific antibody. Inset shows the Western blot analysis of the wild type and CLTC-deficient (KO) cell lines for the presence of CltC.
