# Supplementary material for: Unique features in the intracellular transport of typhoid toxin revealed by a genome-wide screen
Source: PLoS Pathog. 2019 Apr 5;15(4):e1007704. doi: 10.1371/journal.ppat.1007704 (PMC6469816; doi:10.1371/journal.ppat.1007704)
Supplement: S3 Table — (PDF) [file ppat.1007704.s005.pdf]

Supplementary Table 3. List of primers used in this study

Primer	Sequence
CRISPR-F1	AATGGACTATCATATGCTTACCGTAACTTGAAAGTATTTCG
CRISPR-R1	CTTTAGTTTGTATGTCTGTTGCTATTATGTCTACTATTCTTTCC
CRISPR-F2	AATGATACGGCGACCACCGAGATCTACACTCTTTCCCTACACGACGCTCTTCCGATCTtcttgtggaaaggacgaaacaccg
CRISPR-R2-I	CAAGCAGAAGACGGCATACGAGATCGTGATGTGACTGGAGTTCAGACGTGTGCTCTTCCGATCTtctactattctttcccctgcactgt
CRISPR-R2-II	CAAGCAGAAGACGGCATACGAGATACATCGGTGACTGGAGTTCAGACGTGTGCTCTTCCGATCTtctactattctttcccctgcactgt
CRISPR-R2-III	CAAGCAGAAGACGGCATACGAGATGCCTAAGTGACTGGAGTTCAGACGTGTGCTCTTCCGATCTtctactattctttcccctgcactgt
CRISPR-R2-IV	CAAGCAGAAGACGGCATACGAGATTGGTCAGTGACTGGAGTTCAGACGTGTGCTCTTCCGATCTtctactattctttcccctgcactgt
CRISPR-R2-V	CAAGCAGAAGACGGCATACGAGATTTGACTGTGACTGGAGTTCAGACGTGTGCTCTTCCGATCTtctactattctttcccctgcactgt
CRISPR-R2-VI	CAAGCAGAAGACGGCATACGAGATCGAACTGTGACTGGAGTTCAGACGTGTGCTCTTCCGATCTtctactattctttcccctgcactgt
CRISPR Sequencing primer	TCTTCCGATCTCTTGTGGAAAGGACGAAACACCG
sg-VPS51	TGTGTGCGGCTGCGCACCTT
VPS51-screen primer 1	CAAGATCAGCCTGAAGACGC
VPS51-screen primer 2	GTAGAGCTGCAGAAAGTGGC
sg-VPS54	CTTGAGCAGATCAAACCACT
VPS54-screen primer 1	TGTGAAGCTTGCAGATCAGA
VPS54-screen primer 2	TGTTGCCTTCACTCTCTGTAG
sg-COG1	TGCGTCTCGAAAAGAGCCGC
COG1-screen primer 1	CTGAAGCGGCTGGATCTG
COG1-screen primer 2	CTCCTCCTTCTTGTGCTCGA
sg-COG5	TGCTGCTGACGACATCAACC
COG5-screen primer 1	GATTCTGCGTCACCACCTG
COG5-screen primer 2	CTCCGAGGCCAGCTACAG
sg-TMED2	CGACAACACAAACAGCAGAG
sg-SEL1L	GCAGAAATGATGTATCAAAC
SEL1L-screen primer 1	TGAAACTGAAGAAGAGGCTGC
SEL1L-screen primer 2	TTTGGCTTTTCTTATTGCTTCCA
sg-SYVN1	GTATGCCATCCTGATGACGA
SYVN1-screen primer 1	ATCCTGATGACGATGGTGCT
SYVN1-screen primer 2	AGAGCATGTACACAGCCTTG
sg-YKT6	AATTCTCCAAGCAAGTCGAC
YKT6-screen primer 1	GACAATGAATACCCATCCCGG
YKT6-screen primer 2	AGGGCTGGGTAATGGATTGT
sg-YIPF5	TGCGCTTTGTTATATGGAGT
YIPF5-screen primer 1	TAGCCATGGAAGGACAGCAA
YIPF5-screen primer 2	TTGTACATCTGGCCCACTGA
sg-SCYL1	TCACAGCCTCTGTCACGACG
SCYL1-screen primer 1	TGATAACCCTGTGTCCCCTT
SCYL1-screen primer 2	AGATCTCCAGCTCCTTCAGG
sg-Clathrin heavy chain	TCGTTTTCAGGAGCATCTCC
