# Supplementary material for: Unique features in the intracellular transport of typhoid toxin revealed by a genome-wide screen
Source: PLoS Pathog. 2019 Apr 5;15(4):e1007704. doi: 10.1371/journal.ppat.1007704 (PMC6469816; doi:10.1371/journal.ppat.1007704)
Supplement: S4 Table — (PDF) [file ppat.1007704.s006.pdf]

Table S4. Plasmids used in these studies.

Plasmid Name	Source	Reference
Human GeCKOv2 libraries	Addgene	Cat. #1000000049
psPAX2	Addgene	Cat. #12260
lentiCas9-Blast	Addgene	Cat. #52962
pSB5831 px459-*vps51*	Galan Laboratory	This study
pSB5832 px459-*vps54*	Galan Laboratory	This study
pSB5833 px459-*cog1*	Galan Laboratory	This study
pSB5834 px459-*cog5*	Galan Laboratory	This study
pSB5835 px459-*tmed2*	Galan Laboratory	This study
pSB5836 px459-*sel1l*	Galan Laboratory	This study
pSB5837 px459-*syvn1*	Galan Laboratory	This study
pSB5838 px459-*ykt6*	Galan Laboratory	This study
pSB5839 px459-*yipf5*	Galan Laboratory	This study
pSB5840 px459-*yipf6*	Galan Laboratory	This study
pSB5840 sg-CLTC	Galan Laboratory	This study
pC4-Myc-SNAP-GalT	Dr. James Rothman's Laboratory	This study
